# Chronicity of high and low level mupirocin resistance in *Staphylococcus aureus* from 30 Indian hospitals

**DOI:** 10.1038/s41598-023-37399-0

**Published:** 2023-06-22

**Authors:** Rajni Prakash, Amar Garg, Riteshkumar Arya, R. K. Kumawat

**Affiliations:** 1 School of Biological Engineering and Life Sciences, Sobhit Deemed University, Meerut, Uttar Pradesh India; 2 Department of Microbiology, Mehsana Urban Institute of Sciences, Ganpat University, Mehsana, Gujarat India; 3 DNA Division, State Forensic Science Laboratory, Jaipur, Rajasthan India

**Keywords:** Antimicrobials, Bacteriology, Clinical microbiology

## Abstract

Mupirocin is one of the most effective topically used antibiotic for the treatment of dermatitis, nasal carriage, decolonization of methicillin susceptible *Staphylococcus aureus* and eradication of methicillin resistant *Staphylococcus aureus*. Extensive use of this antibiotic has resulted in mupirocin resistance in *Staphylococcus aureus* which is a matter of concern. This study was conducted to evaluate the high and low level of mupirocin resistance in *Staphylococcus aureus* collected from various Indian hospitals. A total of 600 samples, of which 436 were pus specimens and 164 wound site swabs were collected from 30 Indian hospitals. Disc diffusion and agar dilution methods were used to test mupirocin susceptibility in methicillin resistant *Staphylococcus aureus*. Out of 600 *Staphylococcus aureus* isolates, 176 isolates (29.33%) were found to be methicillin resistant *Staphylococcus aureus* (MRSA). Out of 176 non-duplicate MRSA strains, 138 isolates were found to be mupirocin sensitive, 21 isolates had high level resistance whereas 17 isolates had low level resistance to mupirocin, which contributed 78.41%, 11.93% and 9.66% respectively. Multidrug resistant susceptibility was tested for all the MRSA with Cefuroxime, Cotrimoxazole and Vancomycin antibiotics. All the high and low level resistant strain were subjected to genome screening for mupA ileS gene respectively. mupA gene was found positive in all the high level resistant strain and out of 17 low level resistant strain, 16 strain were found point mutation in V588F of ileS gene. Overall, high rate of mupirocin resistance was found in the studied samples which might be a result of indiscriminate use of mupirocin in the population of studied region. This data emphasizes the urgent need for formulation of a well-defined and regulated guidelines for mupirocin use. Moreover, continuous surveillance is needed for the use of mupirocin and routine test should be performed to detect MRSA in patients and health care personnel to prevent MRSA infections.

## Introduction

**Figure 1:**
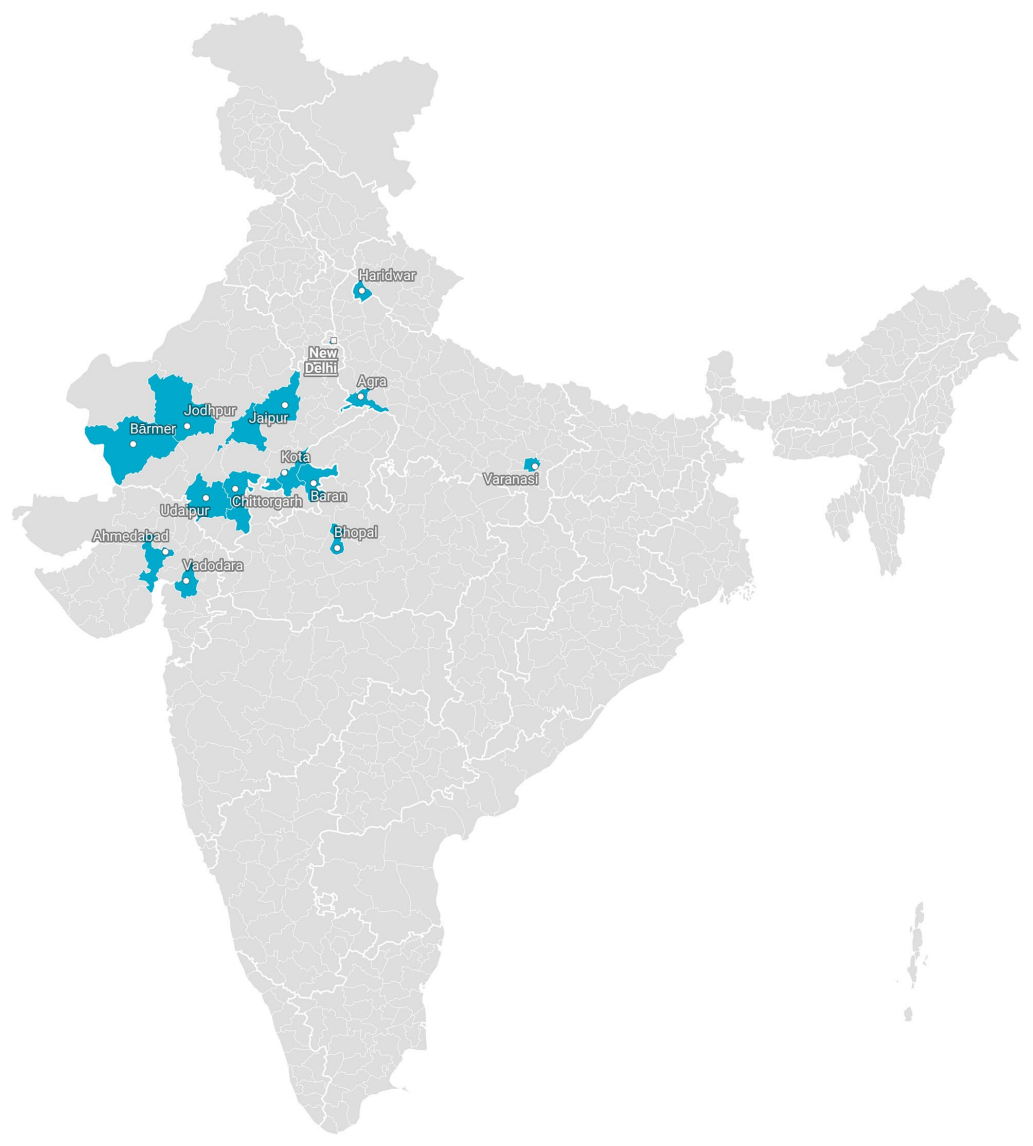
Geographical area of sample collection (Map was created using datasets through https://www.datawrapper.de).

*Staphylococcus aureus* is the most common gram-positive pathogenic bacteria causing skin and soft tissue infections^1^. Its infection severity varies from mild to moderate and is one of the leading cause of high morbidity and mortality rates across the globe^2–4^. Initially ruthless use of penicillin antibiotic for the treatment of various infectious diseases, led to the development of resistance to β-lactams in *Staphylococcus aureus*. In 1960, first methicillin resistant *Staphylococcus aureus* (MRSA) strain was identified. It had lower affinity towards β-lactams due to the modified penicillin binding protein 2a (PBP2a) encoded by mecA gene^5–7^. Mupirocin (pseudomonic acid A) was identified as abroad-spectrum effective antibiotic for gram positive bacteria. This antibiotic inhibited protein synthesis in bacteria through competitively binding with bacterial leucine specific t-RNA aminoacyl synthetase^2,8–10^. Mupirocin was extracted as a secondary metabolite from *Pseudomonas fluorescens* in 1971 and it became the first choice of the entire health service provider as a promising antibiotics since 1976^9–11^. Thereafter, this antibiotic was widely used for the treatment of primary and secondary infections caused by *Staphylococcus aureus* or MRSA and resulted in 80% recovery and 90% eradication of *Staphylococcus aureus*^12^. Usually, mupirocin is prescribed for topical application 2–4 times a day for 5 days and sometimes multiple doses are required to treat the nasal carriage caused by MRSA^13–16^. Overall, long term usage and multiple doses of the antibiotic ledto the development of mupirocin resistance in *Staphylococcus aureus*^16^. Various level of resistance in *Staphylococcus aureus* have been reported worldwide which is matter of concern globally^17–23^. Several studies have been undertaken to explore mupirocin resistance and it rates in *Staphylococcus aureus,* but none are conclusive. Therefore, there is an urgent need to conduct studies which can explore the actual resistance rate worldwide based on which global regulations and standard guidelines could be prepared for the use of mupirocin. These guidelines might regulate the ruthless use of mupirocin and thereby, the developing resistance. However, some studies on mupirocin resistance in *Staphylococcus aureus* have been reported from Indian hospitals but these could not be considered conclusive with small sample size, as India is the second most populous country^23–28^. Keeping this in view, present study was conducted which showed high and low level mupirocin resistance in *Staphylococcus aureus* in Central and North-west India (Fig. 1).

## Results

**Figure 2:**
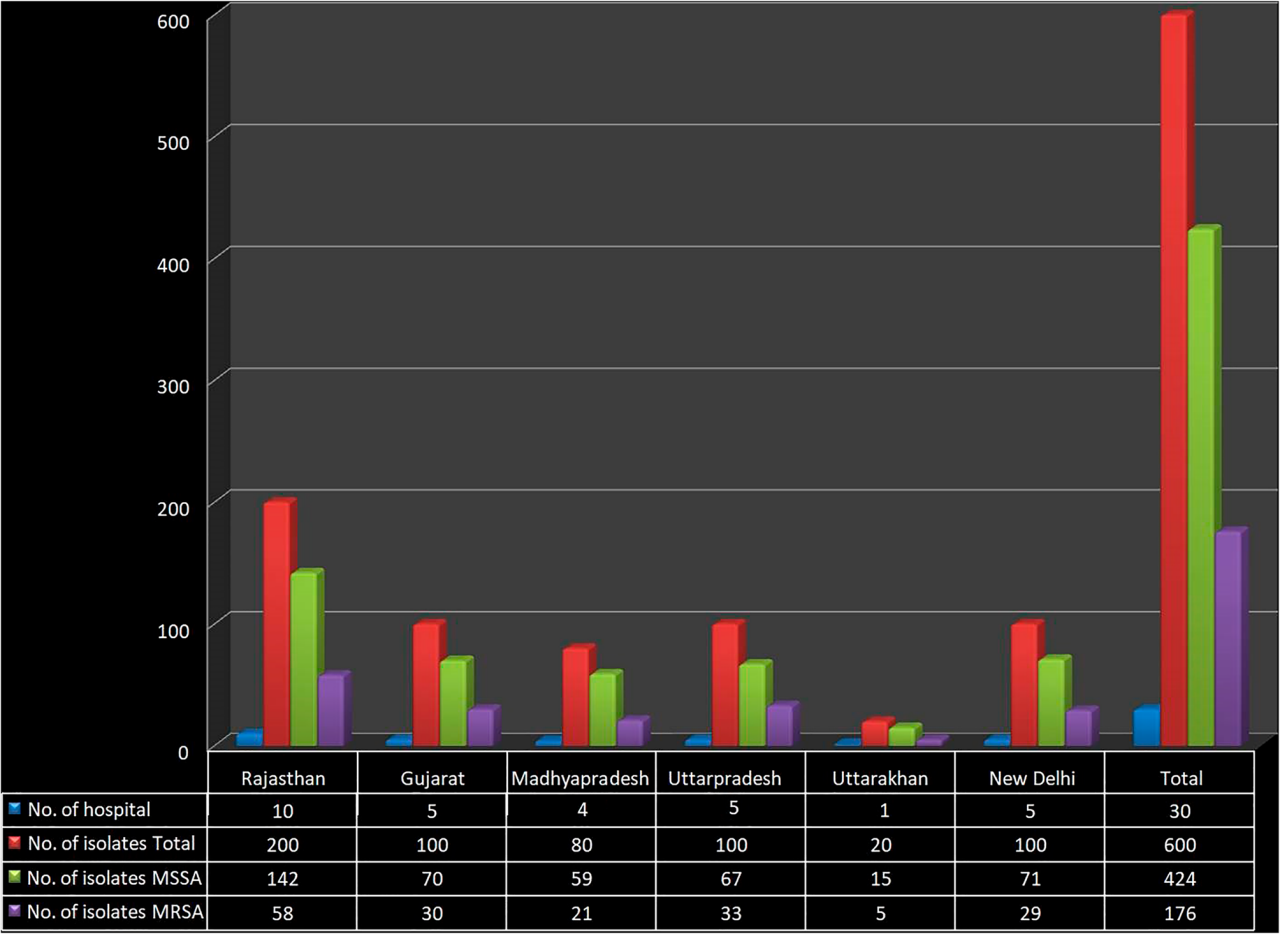
Distribution of sample selection site and mupirocin resistance in methicillin susceptible *Staphylococcus aureus* (MSSA) and methicillin resistant *Staphylococcus aureus* (MRSA).

*Staphylococcus aureus* bacterial strains were detected in all the selected samples. Out of 600 isolates of *Staphylococcus aureus* collected, 424 (70.66%) were found to be methicillin susceptible *Staphylococcus aureus* (MSSA) and 176 (29.33%) were found to be MRSA. Of the 436 pus swabs, 299 (68.58%) were found to be MSSA and 137 (31.42%) were MRSA. Similarly, out of the 164 wound site swabs, 125 (76.22%) were found to be MSSA and 39 (23.78%) were MRSA (Table 1). Among all the *Staphylococcus aureus* isolates, 23% isolates were found to be mupirocin sensitive, 2.83% low level mupirocin resistance and 3.5% high level mupirocin resistance (Table 2). Based on the geographical areas from which samples were collected, in the samples collected from Rajasthan, out of 200 samples, 142 were found to be MSSA and 58 were MRSA, in the samples collected from Gujarat out of 100 samples, 70 were found to be MSSA and 30 were MRSA. Further, in the samples collected from Madhya Pradesh, out of 80 samples, 59 were found to be MSSA and 21 were MRSA, in the samples collected from Uttar Pradesh, out of 100 samples, 67 were found to be MSSA and 33 were MRSA, in the samples collected from Uttarakhand, out of 20 samples, 15 were MSSA and 5 were MRSA, and in the samples collected from New Delhi, out of 100 samples, 71 were found to be MSSA and 29 were MRSA (Table 2) and (Fig. 2). The MRSA isolates obtained were subjected to both disc diffusion and agar dilution methods for assessing mupirocin resistance and both the methods showed similar results. Out of 176 MRSA strain, 138 isolates having MICs ≤ 4 μg/mL were considered as mupirocin sensitive (MuS), 17 isolates with MICs ranging from 8 to 256 μg/mL were considered as low level mupirocin resistance (MuLR), and 21 isolates with MICs ≥ 512 μg/mL were considered as high level mupirocin resistance (MuHR) (Table 2). Based on the geographical region, the identified MRSA strains and MuS, MuLR and MuHR have been shown in Table 2. Highest MuHR (15.15%) was observed in the MRSA strains from Uttar Pradesh region and lowest MuLR (9.5%) in MRSA strain from Madhya Pradesh region (Table 2). Multidrug resistant susceptibility to the MRSA were found to resistant to Cotrimoxazole 64.20% (n = 113), Cefuroxime 46% (n = 81) and Vancomycin 100% (n = 176).

**Table 1:** Distribution of mupirocin resistance in isolates of *Staphylococcus aureus* in relation to MSSA, MRSA and site of isolation.

Site of isolation	No. of bacterial isolates	No. (%) MSSA	No. (%) MRSA
Pus specimen	436	299 (68.58%)	137 (31.42%)
wound swab	164	125 (76.22%)	39 (23.78%)
Total	600	424 (70.66%)	176 (29.33%)

*MSSA* methicillin sensitive *Staphylococcus aureus*, *MRSA* methicillin resistant *Staphylococcus aureus.*

**Table 2:** Distribution of low and high level mupirocin resistance in methicillin susceptible *Staphylococcus aureus* (MSSA) and methicillin resistant *Staphylococcus aureus* (MRSA).

State	No. of hospital	No. of isolates			Mupirocin resistance sensitive MRSA (MIC range ≤ 4 μg/mL)	Low level mupirocin resistance sensitive MRSA (MIC range 8–256 μg/mL)	High level mupirocin resistance sensitive MRSA (MIC range ≥ 512 μg/mL)
		Total	MSSA	MRSA	No. of isolates	No. of isolates	No. of isolates
Rajasthan	10	200	142 (71%)	58 (29%)	47 (81.03%)	5 (8.62%)	6 (10.34%)
Gujarat	5	100	70 (70%)	30(30%)	23 (76.66%)	4 (13.33%)	3 (10%)
Madhyapradesh	4	80	59 (73.75%)	21 (26.25%)	17 (80.95%)	2 (9.5%)	2 (9.5%)
Uttarpradesh	5	100	67 (67%)	33 (33%)	25 (75.76%)	3 (9.09%)	5 (15.15%)
Uttarakhand	1	20	15 (75%)	5 (25%)	3 (60%)	1 (20%)	1 (20%)
New Delhi	5	100	71 (71%)	29 (29%)	23 (79.31%)	2 (6.9%)	4 (13.79%)
Total	30	600	424 (70.66%)	176 (29.33%)	138 (78.41%)	17 (9.66%)	21 (11.93%)

### Genome screening of high and low level mupirocin resistant *Staphylococcus aureus* strain

All the identified high and low level mupirocin resistant *Staphylococcus aureus* strain 21 and 17 respectively were screened at gnomic level using mupA gene for high level and ileS gene for low level resistance. All the high level resistant *S.aureus* strain (n = 21) were found to be positive for the mupA gene and this gene was not detected in all the low level mupirocin resistant *S.* *aureus* bacterial strain. Out of 17 low level resistant strain, 16 strain were found mutation in ileS gene at V588F, which contribute about 94% of low level mupirocin resistant *S. aureus* bacteria.

## Discussion

Long term use of mupirocin for the treatment and eradication of MRSA colonization in patients and health care personnel has led to the development of mupirocin resistance and is a matter of great concern^9^. Nowadays, mupirocin is the first choice of clinicians for the treatment of MRSA causing severe infections viz., skin and soft tissue infection, septicemia, pneumonia^29^. Long term hospitalization, direct or indirect contact of body parts like nasal passage and hand with bacteria are the lead risk factor for MRSA infection in patients and health care personnel^1,9^. Various reported data of MRSA have indicated that MRSA related risk management is in worse situation. In the present study, out of 600 *Staphylococcus aureus* isolates obtained from various hospitals, 176 isolates were identified as MRSA, which contributed 29.33% of the total isolates identified. This percentage is quite high from the previously reported studies from India^25–27,30^. This might be due to the comparatively higher exposure of mupirocin in the region from which isolates were collected. In 1991, a study from UK showed 0.23% *Staphylococcus aureus* isolates to be mupirocin resistance out of429 bacterial isolates included in the study^31^. McNeil et al.^32^ showed presence of 9.8% mupirocin resistance MRSA in a study of *Staphylococcus aureus* related to skin and soft tissue infections in the children of USA^32^. In 1998, a study conducted to evaluate the prevalence of high and low level mupirocin resistance from 19 European hospitals showed 1.6% high level resistance and 2.3% low level resistance present among all the *Staphylococcus aureus* isolates included in the study^33^. Some another global studies on mupirocin resistance calculated this to be in the range of 2.5 to 16%, which might be the result of overall increased exposure of mupirocin in the particular geographical region^34–39^.

Similarly, a study conducted from India by *Gadepalli* et al.^28^ showed 5% high level and 1% low level mupirocin resistance among 200 *Staphylococcus aureus* isolates^28^. Chaturvedi, et al.^27^ showed 82 MRSA isolates among 361 *Staphylococcus aureus* which contribute 22.71% of the total isolates collected. Out of 82 MRSA, 8 isolates showed high level mupirocin resistance and 7 isolates showed low level resistance which was 9.76 and 8.54% of total MRSA respectively^27^. Rudresh et al.^26^*,* also showed 8.2% high level and 17% low level mupirocin resistance among 98 *Staphylococcus aureus* isolates tested^26^. Few other studies from India, also have shown 10 to 15% high level and 5 to 10% low level mupirocin resistance^25–27,30^. In the present study, among 176 isolates which were MRSA, 138 isolates were found to be MuRS which contributed 78.41% of the total MRSA and 17 isolates were MuLR which were 9.66% and 21 isolates were MuHR which were 11.93% of the total MRSA isolates. Among all the 600 *Staphylococcus aureus* isolates, 23% isolates were found to be mupirocin sensitive, 2.83% low level mupirocin resistance and 3.5% high level mupirocin resistance. Multidrug resistant susceptibility testing, Cotrimoxazole 64.20% (n = 113), Cefuroxime 46% (n = 81) and Vancomycin 100% (n = 176). Thus, vancomycin was found uniformly resistance for all the identified MRSA^27^.

In genome screening, both high level and low level mupirocin antibiotic resistant S. aureus bacteria were found positive for mupA gene and point mutation at v588f in ileS gene respectively. Various studies have been undertaken, to understand the mechanism of antibiotic tolerance in *Staphylococcus aureus* bacterial strain^40–42^. These studies explored the best possible mechanism of drug resistance in low level antibiotic resistance using ileS gene mapping. Wild type and antibiotic treated *Staphylococcus aureus* strain were subjected for gene sequencing and evaluated for regulation of gene expression. The antibiotic exposed bacterial strains were found up regulation in ileS gene expression. In the up regulation of gene expression was evaluated through binding affinity between Isoleucyl-tRNA synthetase (ileRS) and Isoleucyl-tRNA (ile-tRNA). Normally, without antibiotics resistance the affinity of ileRS and ile t-RNA resulted to successful charging ile-tRNA^ile^ and followed the successful protein synthesis for cell wall and reflected into normal cell growth. When this normal condition was exposed to antibiotics which resulted into bacterial death due to lack protein synthesis required for cell wall synthesis. In the another condition, either competitive inhibition for binding affinity between ileRS and ile t-RNA or mutation in ileS gene resulted into reduced and/or unbinding of ileRS and ile-tRNA developed the stress condition due to isoleucine starvation^43^. This condition developed the accumulation of uncharged ile-tRNA on native protein resulted to activate stress regulator gene relA, that promote to express of stress alarmone (p)ppGpp, which shows stringent response^44–46^. This up regulation of ileS gene expression also directly related to the stress alarmone (p)ppGpp stringent response and it is the possible factor to develop antibiotic tolerance in the bacteria^43^^,^^47^^,^^48^. This might be possible due to mutation at V588F of ileS parent gene. Earlier some studies were also found similar genome screening of high level and low level mupirocin antibiotic resistant *S. aureus* bacteria^40–43^.

Overall, the finding of present study was found in concordance with previously reported global and Indian studies and added further information on drawbacks especially increasing antibiotic resistance due to ruthless use of mupirocin.

## Conclusion

Present study showed 11.93% of high level and 9.66% of low-level mupirocin resistance *Staphylococcus aureus* among all the MRSA isolates. This data could be of serious concern among clinicians to manage the growing challenge of MRSA. To overcome this concern, ruthless use of mupirocin and it’s over the counter sale should be regulated. Overall, 29.33% MRSA was detected and out of these isolates, strains were found to have high- and low-level mupirocin resistance. Among 38 high- and low-level resistant strains, 55.26% isolates showed high level and 44.74% isolates showed low level resistance, which might be result of much higher exposure to mupirocin. The findings of the present study could serve as basic information which necessitates the development of well-defined and regulated guidelines along with continuous surveillance for the use of mupirocin. Moreover, routine tests should also be made mandatory for the detection of MRSA in patients and health care personnel to control MRSA infections.

## Materials and methods

### Sample selection

Based on most possible clinical symptoms of *Staphylococcus aureus* infections, a total of 600 samples were collected. Of these, 436 were pus specimen and 164 were wound site swabs collected from 30 Indian hospitals of Central and North-west India located in states of Rajasthan, Gujarat, Uttar Pradesh, Uttarakhand Madhya Pradesh and New Delhi (Fig. 1). The experimental protocols were approved by institution ethics committee of Institute for Medical Sciences and Research Centre, Jaipur National University, Jaipur, Rajasthan (ECR/905/Inst/RJ/2017) with approval no. JNUIMSRC/IEC/2018/46. All methods were carried out in accordance with relevant guidelines and regulations of the committee. Also a written informed consent was taken from all the participants of the study. Pus specimen and wound site swabs were collected following the standard protocols and stored under proper condition in transport tubes with labels and transported to the microbiology laboratory for further analysis.

### Culture and identification of *Staphylococcus aureus* species

All the collected samples were cultured in blood agar culture media with incubation at 37 °C for 24 h. Grown colonies of *Staphylococcus aureus* species were identified using standard basic biochemical methods viz., colony morphology, gram staining, coagulase test, sugar fermentation test, ornithine decarboxylase test, urease and acetoin production test, nitrate reduction test and further confirmed using polymerase chain reaction (PCR) method through detection of femB gene^28,44^.

### Isolation and identification of MRSA

As per the recommendations of Clinical and Laboratory Standards Institute (CLSI)^25,45^ antimicrobial susceptibility was tested using disc diffusion and agar dilution methods. All the identified *Staphylococcus aureus* strains were tested for MRSA using 30 µg cefoxitin disc^27,46^. Furthermore, bacterial lawn culture was prepared using Muller-Hinton agar (MHA) and 4% of sodium chloride was used to determine the zone of inhibition after aerobic incubation at 35 °C for 24 h. As per CLSI, if the inhibitory zone is ≥ 22 mm, the strain is considered as sensitive and if the inhibitory zone is ≤ 21 mm, it is considered as resistant. Therefore, bacterial isolates which showed inhibitory zone ≤ 21 mm were considered as MRSA, and were also further confirmed using PCR method.

### Mupirocin susceptibility test

As per the instructions of CLSI, minimum inhibitory concentrations (MICs) for all the bacterial isolates tested for mupirocin resistance were determined using disc diffusion on MHA agar and agar dilution methods.

### Multidrug resistant susceptibility test of mupirocin resistant S. aureus

**Figure 3:**
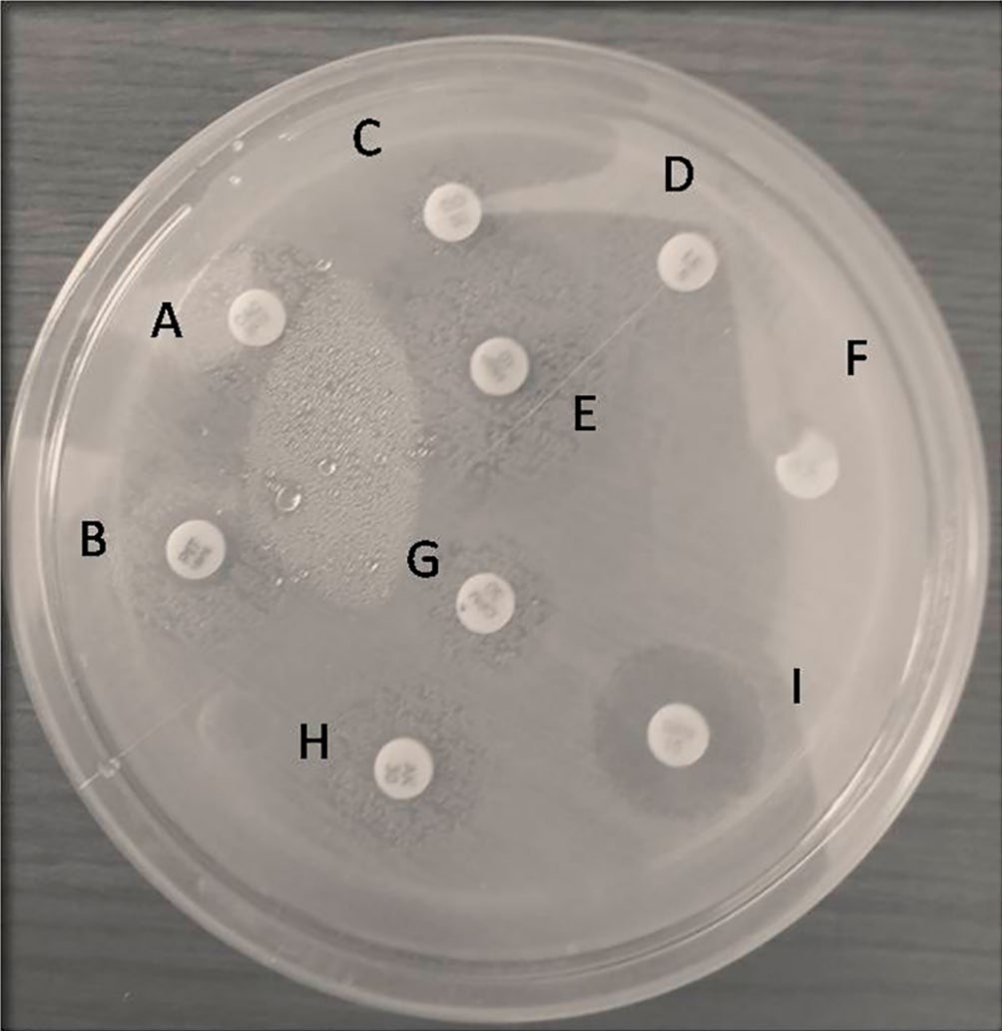
Disk diffusion assay picture for representative multi drug resistant high level mup resistant strain: A,B Mupirocin 5 µg disk; C,D Mupirocin 200 µg disk; E Cotrimoxazole 1.25 µg ; F Cotrimoxazole 23.75 µg; G,H Vancomycin 30µgl; I Cefuroxime 30 µg.

All the MRSA (n = 176) were also tested for antibiotic susceptibility as per the recommended guideline of CLSI using disc diffusion method for the antibiotics cefuroxime (30 µg), Cotrimoxazole (1.25 µg and 23.75 µg) and Vancomycin (30 µg) (Fig. 3) as described elsewhere^27^.

### Disc diffusion method for determination of MICs for Mupirocin

5 µg and 200 µg mupirocin discs were used for determination of MICs for all the bacterial isolates. Both the discs were placed in the bacterial growth plate on MHA agar and incubated at 35 ± 2 °C for 24 h. Zone of inhibition diameter in the plate was carefully examined and measured using transmitted light source. In the bacterial growth plate, if no inhibitory zone was found, it was considered as mupirocin resistant (MuR); if inhibitory zone was found for 5 µg mupirocin discs, it was considered as low level mupirocin resistant (MuLR); and if there was inhibitory zone for both 5 µg and 200 µg mupirocin discs, it was considered as high level mupirocin resistant (MuHR).

### Agar dilution method for determination of MICs for Mupirocin

Mupirocin concentration within the dilution range from 0.016 to 1024 μg/ml was used in MHA agar for the determination of MICs for all the bacterial isolates as per the CLSI guidelines. Isolates with MIC ≥ 512 μg/ml were considered as MuHR, with MICs 8–256 μg/ml were considered as MuLR, and with ≤ 4 μg/ml were considered as mupirocin sensitive (MuS).

### Genome level screening

**Figure 4:**
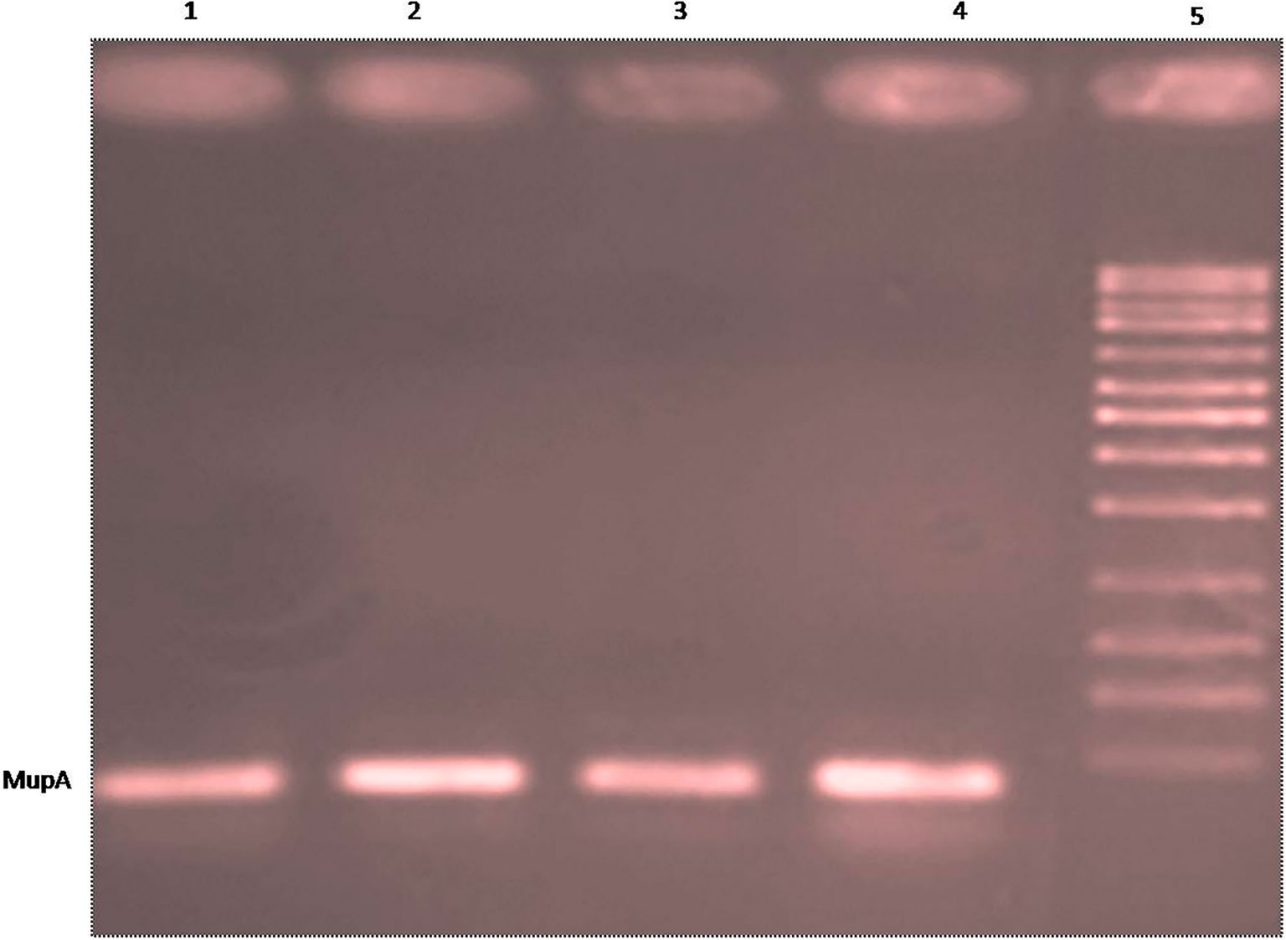
The amplified plasmid mediated Mup A gene of high level mupirocin resistant staphylococcus aureus bacterial strain’s after gel electrophoresis.

All the identified high and low level mupirocin resistant *Staphylococcus aureus* strains were subjected to genome level screening. High level mupirocin resistant *Staphylococcus aureus* bacterial strains were genotyped for plasmid mediated 1650 bp mupA and 674 bp mupB gene. Low level mupirocin resistant *Staphylococcus aureus* bacterial strains were genotyped using primers (Table 3) for 472 pb region of ileS gene using forward and reverse primer sets^41,42^ as described in Table 3. For the amplification of target region of the DNA strand, reaction buffer (2.5µL), Magnesium Chloride (MgCl_2,_ 0.5 µL of 100 mM), Taq DNA polymerase enzyme (2.5 units), dNTPs (0.5 µL of 50 µM) and primer mix (0.5 µL of 10pMol) and template DNA 1 µL were used in PCR cycle using Eppendorf Mastercycler® nexus PCR thermal cycler as the condition shown in Fig. 4. Control *Staphylococcus aureus* strain ATCC25923 was used for the quality assurance of the experiment^43^. DNA sequencing was done using BigDye terminator method (sangar sequencing) via genetic analyzer 3500XL instrument (ThermoFisher Scientific, CA, USA) as per the standard recommendations for the sequencing protocol.

**Table 3:** Primers used in this study.

Target gene	Primer	Primer sequence
MupA	mupA-F	5′-TATATTATGCGATGGAAGGTTGG-3′
	mupA-R	5′-AATAAAATCAGCTGGAAAGTGTTG-3′
ileS	IleS-1	5′-TACCGCGAGCAATCGTCCCT-3′
	IleS-2	5′-TGTTGGCATCGTGGGCATAG-3′

### Statistical analysis

The results were tabulated, and frequency data was statistically analyzed using Microsoft office excel spreadsheet. Chi-square test was performed to determine the significance level and *P* < 0.05 value was considered as statistically significant.

### Ethical approval

The study was approved by institution ethics committee of Institute for Medical Sciences and Research Centre, Jaipur National University, Jaipur, Rajasthan (ECR/905/Inst/RJ/2017) with approval number JNUIMSRC/IEC/2018/46.

## Data Availability

The datasets used and/or analyzed during the current study available from the corresponding author on reasonable request.

## References

[1] Dadashi M, Hajikhani B, Darban-Sarokhalil D, van Belkum A, Goudarzi M (2020). Mupirocin resistance in *Staphylococcus aureus*: A systematic review and meta-analysis. J. Glob. Antimicrob. Resist..

[2] Caffrey AR, Quilliam BJ, LaPlante KL (2010). Risk factors associated with mupirocin resistance in meticillin-resistant *Staphylococcus aureus*. J. Hosp. Infect..

[3] Chen Y (2014). Characterization of *Staphylococcus aureus* from distinct geographic locations in China: An increasing prevalence of spa-t030 and SCC mec type III. PLoS One.

[4] Dadashi M (2018). Methicillin-resistant *Staphylococcus aureus* (MRSA) in Iran: A systematic review and meta-analysis. J. Glob. Antimicrob. Resist..

[5] Hanssen A-M, Ericson Sollid JU (2006). SCC mec in *Staphylococci*: Genes on the move. FEMS Immunol. Med. Microbiol..

[6] Chaves F, García-Martínez J, de Miguel S, Otero JR (2004). Molecular characterization of resistance to mupirocin in methicillin-susceptible and-resistant isolates of *Staphylococcus aureus* from nasal samples. J. Clin. Microbiol..

[7] Lim KT, Yeo CC, Suhaili Z, Thong KL (2012). Comparison of methicillin-resistant and methicillin-sensitive *Staphylococcus aureus* strains isolated from a tertiary hospital in Terengganu, Malaysia. Jpn. J. Infect. Dis..

[8] Hetem DJ, Bonten MJM (2013). Clinical relevance of mupirocin resistance in *Staphylococcus aureus*. J. Hosp. Infect..

[9] Khoshnood S (2019). A review on mechanism of action, resistance, synergism, and clinical implications of mupirocin against *Staphylococcus aureus*. Biomed. Pharmacother..

[10] Sutherland R (1985). Antibacterial activity of mupirocin (pseudomonic acid), a new antibiotic for topical use. Antimicrob. Agents Chemother..

[11] Sutherland, R. Pseudomonic acid, an antibiotic produced by Pseudomonas fluorescens. In *Proceedings of the 16th Interscience Conference on Antimicrobial Agents and Chemotherapy* (1976).

[12] Dutta P, Das S (2016). Mammalian antimicrobial peptides: Promising therapeutic targets against infection and chronic inflammation. Curr. Top. Med. Chem..

[13] Eltringham I (1997). Mupirocin resistance and methicillin-resistant *Staphylococcus aureus* (MRSA). J. Hosp. Infect..

[14] Hudson IRB (1994). The efficacy of intranasal mupirocin in the prevention of *Staphylococcal* infections: A review of recent experience. J. Hosp. Infect..

[15] Schmitz F-J, Jones ME (1997). Antibiotics for treatment of infections caused by MRSA and elimination of MRSA carriage. What are the choices?. Int. J. Antimicrob. Agents.

[16] Abbasi-Montazeri E (2013). The prevalence of methicillin resistant *Staphylococcus aureus* (MRSA) isolates with high-level mupirocin resistance from patients and personnel in a burn center. Burns.

[17] Shittu AO, Udo EE, Lin J (2009). Phenotypic and molecular characterization of *Staphylococcus aureus* isolates expressing low-and high-level mupirocin resistance in Nigeria and South Africa. BMC Infect. Dis..

[18] Desroches M (2013). Prevalence of mupirocin resistance among invasive coagulase-negative *staphylococci* and methicillin-resistant *Staphylococcus aureus* (MRSA) in France: Emergence of a mupirocin-resistant MRSA clone harbouring mupA. J. Antimicrob. Chemother..

[19] Coombs GW (2013). Australian group on antimicrobial resistance hospital-onset *Staphylococcus aureus* surveillance programme annual report, 2011. Commun. Dis. Intell. Q. Rep..

[20] Jones JC (2007). Mupirocin resistance in patients colonized with methicillin-resistant *Staphylococcus aureus* in a surgical intensive care unit. Clin. Infect. Dis..

[21] Gilbart J, Perry CR, Slocombe B (1993). High-level mupirocin resistance in *Staphylococcus aureus*: Evidence for two distinct isoleucyl-tRNA synthetases. Antimicrob. Agents Chemother..

[22] Park SY, Kim SM, Park SD (2012). The prevalence, genotype and antimicrobial susceptibility of high-and low-level mupirocin resistant methicillin-resistant *Staphylococcus aureus*. Ann. Dermatol..

[23] Jayakumar S, Meerabai M, Shameem Banu AS, Mathew R, Kalyani R (2013). Prevalence of high and low level mupirocin resistance among *staphylococcal* isolates from skin infection in a tertiary care hospital. J. Clin. Diagn. Res. JCDR.

[24] Perumal N, Murugesan S, Ramanathan V, Krishnan P (2016). High occurrence of high-level mupirocin & chlorhexidine resistant genes in methicillin resistant *Staphylococcal* isolates from dialysis unit of a tertiary care hospital. Indian J. Med. Res..

[25] Agarwal L, Singh AK, Sengupta C, Agarwal A (2015). Nasal carriage of methicillin-and mupirocin-resistant S. aureus among health care workers in a tertiary care hospital. J. Res. Pharm. Pract..

[26] Rudresh MS (2015). Prevalence of mupirocin resistance among Staphylococci, its clinical significance and relationship to clinical use. J. Lab. Physicians.

[27] Chaturvedi P, Singh AK, Shukla S, Agarwal L (2014). Prevalence of mupirocin resistant *Staphylococcus aureus* isolates among patients admitted to a tertiary care hospital. N. Am. J. Med. Sci..

[28] Gadepalli R (2007). Mupirocin resistance in *Staphylococcus aureus* in an Indian hospital. Diagn. Microbiol. Infect. Dis..

[29] Gordon RJ, Lowy FD (2008). Pathogenesis of methicillin-resistant *Staphylococcus aureus* infection. Clin. Infect. Dis..

[30] Joshi S (2013). Methicillin resistant *Staphylococcus aureus* (MRSA) in India: Prevalence & susceptibility pattern. Indian J. Med. Res..

[31] Wise R, Johnson J (1991). Mupirocin resistance. Lancet.

[32] McNeil JC, Hulten KG, Kaplan SL, Mason EO (2014). Decreased susceptibilities to retapamulin, mupirocin, and chlorhexidine among *Staphylococcus aureus* isolates causing skin and soft tissue infections in otherwise healthy children. Antimicrob. Agents Chemother..

[33] Schmitz FJ (1998). The prevalence of low-and high-level mupirocin resistance in *staphylococci* from 19 European hospitals. J. Antimicrob. Chemother..

[34] Warren DK (2016). Prevalence of qacA/B genes and mupirocin resistance among methicillin-resistant *Staphylococcus aureus* (MRSA) isolates in the setting of chlorhexidine bathing without mupirocin. Infect. Control Hosp. Epidemiol..

[35] Nikfar G, Jamali H, Ahmadzadagan J (2016). Simultaneous detection of methicillin-and mupirocin-resistant genes in *Staphylococcus aureus* isolated with multiplex PCR from patients in the city of Darab, Fars Province, Iran. Int. J. Med. Res. Heal. Sci..

[36] Nezhad RR, Meybodi SM, Rezaee R, Goudarzi M, Fazeli M (2017). Molecular characterization and resistance profile of methicillin resistant *Staphylococcus aureus* strains isolated from hospitalized patients in intensive care unit, Tehran-Iran. Jundishapur J. Microbiol..

[37] Joshi PR (2017). Emergence of *staphylococcal*
*cassette* chromosome mec type I with high-level mupirocin resistance among methicillin-resistant *Staphylococcus aureus*. Asian Pac. J. Trop. Biomed..

[38] Shittu AO, Lin J (2006). Antimicrobial susceptibility patterns and characterization of clinical isolates of *Staphylococcus aureus* in KwaZulu-Natal province, South Africa. BMC Infect. Dis..

[39] Rotger M, Trampuz A, Piper KE, Steckelberg JM, Patel R (2005). Phenotypic and genotypic mupirocin resistance among *staphylococci* causing prosthetic joint infection. J. Clin. Microbiol..

[40] Singh M (2020). RNA sequencing identifies a common physiology in vancomycin-and ciprofloxacin-tolerant *Staphylococcus*
*aureus* induced by ileS mutations. Antimicrob. Agents Chemother..

[41] Singh M (2017). In vitro tolerance of drug-naive *Staphylococcus*
*aureus* strain FDA209P to vancomycin. Antimicrob. Agents Chemother..

[42] Matsuo M (2019). Genetic and transcriptomic analyses of ciprofloxacin-tolerant *Staphylococcus*
*aureus* isolated by the replica plating tolerance isolation system (REPTIS). Antimicrob. Agents Chemother..

[43] Reiß S (2012). Global analysis of the *Staphylococcus*
*aureus* response to mupirocin. Antimicrob. Agents Chemother..

[44] Grundy FJ (1997). The *Staphylococcus*
*aureus* ileS gene, encoding isoleucyl-tRNA synthetase, is a member of the T-box family. J. Bacteriol..

[45] Iaccarino M, Berg P (1971). Isoleucine auxotrophy as a consequence of a mutationally altered isoleucyl-transfer ribonucleic acid synthetase. J. Bacteriol..

[46] Geiger T (2010). Role of the (p) ppGpp synthase RSH, a RelA/SpoT homolog, in stringent response and virulence of *Staphylococcus*
*aureus*. Infect. Immun..

[47] Abranches J (2009). The molecular alarmone (p) ppGpp mediates stress responses, vancomycin tolerance, and virulence in *Enterococcus*
*faecalis*. J. Bacteriol..

[48] Geiger T, Kästle B, Gratani FL, Goerke C, Wolz C (2014). Two small (p) ppGpp synthases in *Staphylococcus*
*aureus* mediate tolerance against cell envelope stress conditions. J. Bacteriol..

